# Experiences of severe childhood maltreatment, depression, anxiety and alcohol abuse among adults in Finland

**DOI:** 10.1371/journal.pone.0177252

**Published:** 2017-05-08

**Authors:** Wail Rehan, Jan Antfolk, Ada Johansson, Patrick Jern, Pekka Santtila

**Affiliations:** 1 Department of Psychology, Åbo Akademi University, Turku, Finland; 2 Department of Psychology and Speech-Language Pathology, University of Turku, Turku, Finland; University of New South Wales, AUSTRALIA

## Abstract

Childhood maltreatment increases the risk of subsequent depression, anxiety and alcohol abuse, but the rate of resilient victims is unknown. Here, we investigated the rate of victims that do not suffer from clinical levels of these problems after severe maltreatment in a population-based sample of 10980 adult participants. Compared to men, women reported more severe emotional and sexual abuse, as well as more severe emotional neglect. For both genders, severe emotional abuse (*OR* = 3.80 [2.22, 6.52]); severe physical abuse (*OR* = 3.97 [1.72, 9.16]); severe emotional neglect (*OR* = 3.36 [1.73, 6.54]); and severe physical neglect (*OR* = 11.90 [2.66, 53.22]) were associated with depression and anxiety while only severe physical abuse (*OR* = 3.40 [1.28, 9.03]) was associated with alcohol abuse. Looking at men and women separately, severe emotional abuse (*OR* = 6.05 [1.62, 22.60] in men; *OR* = 3.74 [2.06, 6.81] in women) and severe physical abuse (*OR* = 6.05 [1.62, 22.60] in men; *OR* = 3.03 [0.99, 9.33] in women) were associated with clinical levels of depression and anxiety. In addition, in women, severe sexual abuse (*OR* = 2.40 [1.10, 5.21]), emotional neglect (*OR* = 4.78 [2.40, 9.56]), and severe physical neglect (*OR* = 9.86 [1.99, 48.93]) were associated with clinical levels of depression and anxiety. Severe emotional abuse in men (*OR* = 3.86 [0.96, 15.48]) and severe physical abuse in women (*OR* = 5.18 [1.48, 18.12]) were associated with alcohol abuse. Concerning resilience, the majority of severely maltreated participants did not report clinically significant levels of depression or anxiety (72%), or alcohol abuse (93%) in adulthood. Although the majority of severely abused or neglected individuals did not show clinical levels of depression, anxiety or alcohol use, severe childhood maltreatment increased the risk for showing clinical levels of psychopathology in adulthood.

## Introduction

Severe child maltreatment is conventionally defined within child protection practice as severe physical, emotional, sexual abuse and/or severe physical and emotional neglect by adults [1]. Severity can be defined on the basis of the type of maltreatment, its frequency, if the child was subjected to multiple forms of maltreatment, if a weapon had been used, if the maltreatment resulted in an injury, and if the abuse was considered severe by the victim. For sexual abuse, even a single experience is often considered to be severe [1].

### Childhood maltreatment and its psychosocial consequences

There are annually over one million victims of childhood maltreatment in the USA alone and childhood maltreatment has a large public health impact [2]. Several studies show that childhood physical, emotional, and sexual abuse are all related to an increased risk of depression and anxiety disorders in adulthood [3–9]. Other studies have found that the severity of abuse and neglect is associated with increased depression and anxiety symptoms in adulthood [10–12]. This means that as a general rule, the more severe the abuse and neglect, the more likely the abused individuals are to show symptoms of depression and anxiety. There is also a robust relationship between childhood maltreatment and later alcohol abuse [13–16]. For example, Young-Wolff et al. [17] found that men who had experienced childhood maltreatment were 1.7 times more likely to suffer from alcohol abuse in adulthood than men who did not report experiences of childhood maltreatment. Similar findings have been made when investigating the consequences of abuse and neglect in women (e.g., [18]).

Findings from a study by Schwandt et al. [19] suggested that the severity of emotional and physical abuse plays a prominent role in the development of alcohol abuse. In line with these results suggesting a role of the severity of childhood abuse on later substance misuse, Hyman et al. [20] found that the severity of abuse was predictive of cocaine use after having been discharged from an inpatient treatment for cocaine addiction. This was true for women but not for men. Kendler et al. [21] showed that women who had experienced child sexual abuse reported higher incidences of alcohol abuse. Twin studies have also shown that childhood sexual abuse increases the risk of alcohol abuse and addiction later in life [21–24]. To summarize, there is a strong, robust relationship between childhood maltreatment and mental disorders in adulthood. These associations include associations between childhood experiences of physical abuse, emotional abuse, and neglect, respectively, and mental disorders such as depression and anxiety disorders, and alcohol abuse [25–26]. Moreover, multi-type maltreatment in childhood is associated with greater impairment in adulthood, and this association also includes a range of psychological and behavioral problems, such as depression, anxiety, and alcohol abuse [27].

However, not all victims of childhood maltreatment develop symptoms of substance abuse or psychopathology in adulthood. Meta-analyses suggest that many (but not all) children who have experienced abuse succeed in overcoming some of the possible negative outcomes [28]. For example, Klika and Herrenkohl [28] found that some individuals who have experiences of abuse in childhood do not suffer long-term negative sequelae. Collishaw et al. [29] reported that despite serious experiences of childhood sexual or physical abuse, some individuals did not develop psychiatric problems during adulthood. Moreover, Hamilton et al. [30] reported that emotional neglect did not significantly predict increases in depressive or anxiety symptoms later in life. It has been estimated that 12–22% of maltreated individuals are functioning well despite experiencing childhood maltreatment [31].

### The current study

Several studies have focused on only experiencing one type of maltreatment (e.g., sexual abuse) or one type of outcome (e.g., depression). Moreover, most previous studies have relied on either convenience samples or samples from health care services, and especially samples of the latter kind might bias the results and show less resilience than is actually the case.

In the present study, we used a large, population-based sample of Finnish men and women. The types of maltreatment included emotional, physical, and sexual abuse as well as emotional and physical neglect.

Thus, the aims of the present study were to:

1. Investigate gender differences in severe experiences of different types of childhood abuse;
2. Compare if and how individuals reporting severe experiences of different types of childhood abuse differ from individuals who did not report experiencing childhood abuse, in terms of presence of clinically significant symptoms of depression and anxiety in adulthood; and
3. Compare if and how individuals reporting severe experiences of different types of childhood abuse differ from individuals who did not report experiencing childhood abuse in terms of presence of alcohol abuse symptoms in adulthood.

## Materials and methods

### Participants

The sample consisted of 3766 male and 7214 female twins and their siblings. The mean age of the men was 29.2 years (*SD* = 7.4), and of the women, 28.8 (*SD* = 7.2) years. The participants were a subset of both the first and the second data collections of the population-based Genetics of Sex and Aggression (GSA) survey conducted in 2005 and 2006 [32]. Of these two data collections, the first data collection included responses from 3,604 individuals (response rate = 36%) and the second data collection included responses from 10,524 individuals (response rate = 45%), totaling 14,126 individuals. For sociodemographic characteristics of the participants see Table 1 (for further information on the data collection, see [32]). Data analyses in the present study were based on a subset of these participants, since 3,146 participants did not provide responses to one or more of the items used. Because the sample consisted of twins, responses regarding the frequency of experiences of childhood maltreatment and sexuality were compared with a different Finnish population-based sample [33] of the general population. We found no significant differences, suggesting that the responses for our sample were generalizable to non-twin individuals. Re-analyzing data from another Finnish sample [33], the frequencies for childhood maltreatment according to our operational definition (see the Measures section) was 1.6% for sexual abuse, 1.1% for physical abuse, 10.6% for emotional abuse, 9.0% for emotional neglect, and 1.3% for physical neglect.

**Table 1:** Sociodemographic characteristics of the participants.

	Men	Women
Age	28.8 (7.2)	29.2 (7.4)
Do you have a regular partner?	67.8%	78.1%
For how long have you been together with your current partner? ^a^	4.21 (1.16)	4.40 (1.16)
Does your mother have a college degree? ^b^	20.3%	15.3%
Does your father have a college degree? ^b^	16.4%	13.9%
Do you have children? ^c^	64.3%	53.6%

^a^ 1.00 less than a month; 2.00 more than a month but less than 6 month; 3.00 7–12 months; 4.00 1–3 years; 5.00 4–10 years; 6.00 more than 10 years

^b^ Missing values varied between 1116–1952 for men and 1868–3022 for women (variable not available for the first data collection).

^c^ Missing values varied between 272 for men and 722 for women due to no responding.

The research and data collection plan for the first data collection was approved by the Departmental Review Board of the Department of Psychology, Abo Akademi University in Turku, Finland, as it did not contain biological samples. The cover letter to the questionnaire indicated that participation was totally voluntary and anonymous. The research plan for the second data collection was approved by the Ethics Committee of the Abo Akademi University. Both ethical reviews were conducted in accordance with the Declaration of Helsinki. All participants provided written informed consent to their voluntary and anonymous participation either via a secure web page or by a paper consent form.

### Measures

#### Childhood maltreatment

The Childhood Trauma Questionnaire (CTQ) [34] was used to assess the degree to which individuals had experienced childhood maltreatment during their whole childhood (an upper age limit was not specified). In the CTQ five types of abuse (emotional, physical, and sexual) and neglect (emotional and physical) are measured by presenting participants with five statements for each type of childhood maltreatment. These items include items such as *“People in my family said hurtful or insulting things to me”* (emotional abuse), *“I was punished with a belt*, *a board*, *a cord*, *or some other hard object”* (physical abuse), “*Someone tried to touch me in a sexual way*, *or tried to make me touch them*” (sexual abuse), “*People in my family looked out for each other*” (emotional neglect), and “*I had to wear dirty clothes*” (physical neglect). Responses to these statements were given on a 0 (“*never*”) to 4 (“*very often*”) Likert-type scale indicating the extent to which participants had experienced abusive and/or neglectful acts before the age of 18. The aggregated scores on each of the CTQ subscales thus range from 0 to 20 with higher scores indicative of greater abuse severity. CTQ has demonstrated good reliability, including test-retest reliability coefficients ranging from .79 to .86 [34]. CTQ has also been shown to have convergent validity with both a clinical-rated interview if childhood abuse and therapists’ ratings of abuse [35]. In our sample, the questionnaire had good internal consistency. The Cronbach’s α was .73 for physical abuse, .82 for emotional abuse, .89 for sexual abuse, .86 for emotional neglect and .56 for physical neglect.

Based on the responses of the participants, we created the following categorizations: severe experiences of abuse consisted of participants who had 15 or more points on CTQ variables (i.e., at least a score of 3 on a scale ranging from 0–4 on each of the five items that form each CTQ subscale).

#### Anxiety and depression

We used the Brief Symptom Inventory-18 BSI-18; [36] to measure symptoms of anxiety and depression that the participants experienced during the preceding seven days. BSI-18 has been shown to have appropriate internal consistency, test-retest reliability, and convergent-discriminant validity [36]. We formed composite variables from items measuring anxiety (e.g., “*Feeling so restless one could not sit still*”) and depression (e.g., “*Feeling hopeless about the future”*) on the basis of the factor structure suggested by [36], with six items in each factor. The response options ranged from 0 (*“not at all”*) to 4 (*“very often”*). We used a value of 9 or higher on this composite variable as indicating clinically significant levels of depression and anxiety, as suggested by the instrument’s author on the basis of psychometrical assessments [36]. In the current sample, Cronbach’s α was .84 for the depression domain, and .85 also for the anxiety domain. Higher scores indicated more psychological distress at the moment of measurement.

#### Alcohol abuse

We used the World Health Organization’s Alcohol Use Disorder Identification Test (AUDIT, [37]) to measure alcohol abuse. The AUDIT is a 10-item (e.g., “*How many drinks containing alcohol do you have on a typical day when you are drinking*?”) self-administered screening instrument for hazardous and harmful alcohol consumption. It covers the areas of alcohol consumption, drinking behavior, and alcohol-related problems. Responses to each item are scored from 0 to 4, yielding a maximum possible score of 40. Individuals are classified as low-risk drinkers (score 1–7); at-risk drinkers (score 8–15); high-risk drinkers (score 16–19); or likely dependent drinkers (score 20–40). We use the cut-off score of 16 or more which has been suggested by WHO to identify a high level of problematic alcohol use. Cronbach’s α was .98 for the AUDIT.

### Statistical analyses

Statistical analyses were performed with the Statistical Package for the Social Sciences (Version 21.0; SPSS. Inc., Chicago, Illinois). We used descriptive statistics (frequencies) to identify the proportion of men and women having suffered severe abuse and neglect in childhood. Crosstabs and chi-square tests were used to determine differences between men and women in experiences of severe childhood abuse, as well as differences in clinical levels of depression, anxiety, or alcohol abuse between individuals having suffered severe childhood abuse or neglect and those without such experiences. A two- sided *p*-value less than .05 was considered significant in all analyses of direct effects.

## Results

### Descriptive results

The proportion of participants with severe experiences of emotional abuse was 0.6% (*n* = 64). The corresponding proportion for severe experiences of physical abuse was 0.2% (*n* = 26) while the proportions for severe experiences of sexual abuse was 0.4% (*n* = 43). For severe experiences of emotional neglect, the proportion was 0.4% (*n* = 44) and for severe experiences of physical neglect 0.1% (*n* = 7). With regard to gender differences in the different types of severe experiences of abuse, Table 2 shows that there were statistically significant differences between men and women in the proportion of individuals with severe experiences of emotional abuse, sexual abuse and emotional neglect. All of these were more prevalent in women. There were no statistical differences between men and women in terms of having severe experiences of physical abuse and physical neglect.

**Table 2:** Differences between men and women in frequencies of severe experiences of different types of maltreatment.

Factors	Proportion of Maltreated Individuals			χ^2^
	Total	Men	Women	
	% (*n*)	% (*n*)	% (*n*)	
CTQ Emotional Abuse	0.6 (64)	0.2 (9)	0.8 (55)	11.70***
CTQ Physical Abuse	0.2 (26)	0.2 (9)	0.2 (17)	0.00
CTQ Sexual Abuse	0.4 (43)	0.1 (2)	0.6 (41)	16.84***
CTQ Emotional Neglect	0.4 (44)	0.2 (7)	0.5 (37)	6.63**
CTQ Physical Neglect	0.1 (7)	0.0 (1)	0.1 (6)	1.25

Note: Values indicate the percentage and the absolute numbers of maltreated individuals. Chi-square values indicate the relative difference between the proportion of maltreatment among women and the proportion of maltreatment among men.

*** *p* < .001,

** *p* < .01,

* *p* < .05

### Depression and anxiety in individuals with experiences of severe maltreatment

We then investigated whether the proportion of individuals having clinical levels of depression and anxiety was higher in individuals with severe experiences of abuse and neglect compared to individuals with less severe (or no) experiences of abuse and neglect. Table 3 shows that, for both genders, severe experiences of emotional and physical abuse and emotional and physical neglect increased the likelihood of suffering from clinical depression or anxiety compared to less severe experiences of the said forms of childhood maltreatment.

**Table 3:** Differences between men and women in severe experiences of different types of maltreatment on clinical cases of depression and anxiety.

Severe Abuse		Men				Women							
		Depression or Anxiety				Depression or Anxiety				Depression or Anxiety			
		Yes (n)	No (n)	χ^2^	OR (95% CI)	Yes (n)	No (n)	χ^2^	OR (95% CI)	Yes (*n*)	No (n)	*χ*^*2*^	OR (95% CI)
CTQ Emotional Abuse	Yes (*n*)	19	45	27.154***	3.80 (2.22, 6.52)	4	5	9.28**	6.05 (1.62, 22.60)	15	40	21.46***	3.74 (2.06, 6.81)
	No (*n*)	1091	9825			439	3318			652	6507		
CTQ Physical Abuse	Yes (*n*)	8	18	12.241***	3.97 (1.72, 9.16)	4	5	9.28**	6.05 (1.62, 22.60)	4	13	4.14*	3.03 (.99, 9.33)
	No (*n*)	1102	9852			439	3318			663	6534		
CTQ Sexual Abuse	Yes (*n*)	8	35	3.428	2.04 (0.94, 4.41)	0	2	0.27	n.c	8	33	5.18*	2.40 (1.10, 5.21)
	No (*n*)	1102	9835			443	3321			659	6514		
CTQ Emotional Neglect	Yes (*n*)	12	32	14.321***	3.36 (1.73, 6.54)	0	7	0.94	n.c	12	25	23.83***	4.78 (2.40, 9.56)
	No (*n*)	1098	9838			443	3316			655	6522		
CTQ Physical Neglect	Yes (*n*)	4	3	17.051***	11.90 (2.66, 53.22)	1	0	7.50**	n.c	3	3	11.89***	9.86 (1.99, 48.93)
	No (*n*)	1106	9867			442	3323			664	6544		

Note: Odds Ratios above 1 indicate higher prevalence of depression and anxiety in individuals experiencing severe forms or maltreatment.

*** *p* < .001,

** *p* < .01,

* *p* < .05, n.c. = not calculable due to at least one cell having zero observations.

In men, severe abuse experiences were significantly associated with increases in the prevalence of clinical depression or anxiety when it came to experiences of severe emotional and physical abuse and physical neglect. No association was observed for severe sexual abuse and severe emotional neglect. For women, severe experiences of all childhood maltreatment types increased the likelihood of suffering from clinical depression or anxiety compared to other lower experiences of maltreatment.

Next, we explored the proportions of both men and women who were resilient to severe experiences of childhood maltreatment with regards to not suffering from clinical levels of depression or anxiety in adulthood. Depending on the abuse type, 55.6% to 100% of men with experiences of severe abuse did not show clinically significant levels of depression or anxiety. For women, 50% to 80.5% did not show clinically significant levels of depression or anxiety.

### Alcohol abuse in individuals with experiences of severe childhood maltreatment

When considering both genders together, severe experiences of physical abuse (vs. less severe experiences of physical abuse) increased the likelihood of suffering from alcohol abuse. There were no associations between severe experiences of childhood emotional and sexual abuse, or emotional and physical neglect on the alcohol abuse (See Table 4).

**Table 4:** Differences between men and women in severe experiences of different types of maltreatment on clinical cases of alcohol abuse.

Severe Abuse		All Participants				Men				Women			
		Problematic Alcohol Use				Problematic Alcohol Use				Problematic Alcohol Use			
		Yes (n)	No (n)	χ^2^	OR (95% CI)	Yes (n)	No (n)	χ^2^	OR (95% CI)	Yes (*n*)	No (n)	*χ*^*2*^	OR (95% CI)
CTQ Emotional Abuse	Yes (*n*)	5	59	0.158	1.20 (0.48, 3.01)	3	6	4.201*	3.86 (0.96, 15.48)	2	53	0.020	0.90 (0.22, 3.73)
	No (*n*)	718	10198			431	3326			287	6872		
CTQ Physical Abuse	Yes (*n*)	5	21	6.776**	3.40 (1.28, 9.03)	2	7	1.013	2.20 (0.46, 10.62)	3	14	8.245**	5.18 (1.48, 18.12)
	No (*n*)	718	10236			432	3325			286	6911		
CTQ Sexual Abuse	Yes (*n*)	1	42	1.273	0.34 (0.05, 2.45)	0	2	0.261	n.c	1	40	0.263	0.60 (0.82, 4.36)
	No (*n*)	722	10215			434	3330			288	6885		
CTQ Emotional Neglect	Yes (*n*)	2	42	0.299	0.68 (0.16, 2.80)	1	6	0.052	1.28 (0.415, 10.66)	1	36	0.164	0.66 (0.91, 4.86)
	No (*n*)	721	10215			433	3326			288	6889		
CTQ Physical Neglect	Yes (*n*)	0	7	0.494	n.c.	0	1	0.130	n.c	0	6	0.251	n.c
	No (*n*)	723	10973			434	3331			289	6919		

Note: Odds Ratios above 1 indicate higher prevalence of problematic alcohol use in individuals experiencing severe forms or maltreatment.

*** *p* < .001,

** *p* < .01,

* *p* < .05, n.c. = not calculable due to at least one cell having zero observations.

When considering each gender separately, severe experiences of emotional abuse were significantly associated with increases in the prevalence of alcohol abuse in men, and as well as severe experiences of physical abuse were significantly associated with increases in the prevalence of alcohol abuse in women.

Again, the next step was to explore the proportions of both men and women who, following experiencing severe maltreatment, displayed resilience with regards to problematic alcohol use. Depending on the abuse type, 66.7% to 100% of men with experiences of severe abuse did not show problematic alcohol use. For women, 82.4% to 100% did not show problematic alcohol use.

To calculate the percentage of individuals who displayed resilience (i.e., did not have any symptoms of depression and anxiety or problematic alcohol abuse), we summed unaffected individuals across all types of severe maltreatment. The sum of unaffected individuals was then divided by the total sample size. Concerning resilience, the majority of participants with experiences of severe maltreatment in childhood did not report clinically significant levels of depression or anxiety (72%), or alcohol abuse (93%) in adulthood.

## Discussion

The present study investigated five types of maltreatment: emotional, physical and sexual abuse, and physical and emotional neglect; and their relationships to depression, anxiety and alcohol abuse. The study used a population-based sample of 10980 participants and used validated measures of experiences of childhood maltreatment, current depression and anxiety, and current alcohol abuse. More particularly, our aim was to investigate gender differences in victims of severe childhood maltreatment, as well as to compare if and how individuals reporting severe experiences of different types of childhood abuse differ from individuals without such severe experiences in terms of presence of clinically significant symptoms of depression, anxiety and alcohol abuse in adulthood.

The present study found that women reported more childhood experiences of severe emotional, sexual abuse and emotional neglect than men. Our findings are inconsistent with the results of those of previous studies indicating that men reported more childhood experiences of abuse than women [3, 38]. However, our results are consistent with findings suggesting that women are more sensitive than men to the effects of experiences abuse in childhood [29]. Compared to another Finnish population based sample, the frequencies of severe abuse were relatively low in our sample. This could be due to samples being obtained at different times, as abuse in Finland has been decreasing [39], or that in the present study the complete CTQ was used: in the study by Albrecht’s et al. [33], only one item per factor was used. The decrease in measurement reliability that follows from removing 80% of the original items might have inflated the estimates in Albrecht’s study [33].

More specifically, our results revealed that, in men, severe experiences of emotional and physical abuse as well as physical neglect were significantly associated with increases in the prevalence of depression and anxiety symptoms. For women, there was an association between all types of severe childhood maltreatment (emotional, physical and sexual abuse, and physical and emotional neglect) with depression and anxiety symptoms in adulthood. These results were consistent with previous literature indicating that physical abuse and/or emotional abuse are related to depression and anxiety disorders [4–5, 40–41]. These findings also corroborate findings from meta-analyses and extend previous reports of severe experiences of abuse or neglect being associated with greater risk of developing depressive and anxiety disorders in adulthood [26].

When we examined each type of maltreatment for associations with alcohol abuse, the results showed that severe emotional abuse was associated with alcohol abuse in men. For women, severe physical abuse emerged as a predictor for problematic alcohol use. This is consistent with research suggesting that childhood experiences of emotional and physical abuse were found to be the primary predictor of alcohol abuse [19, 42]. It is intriguing, however, that there appears to be a gender difference in response to abuse type, with men having a considerably more severe response to emotional abuse in terms of propensity to develop alcoholism later in life. For example, an explanation for why women appear to suffer greater consequences in terms of abusing alcohol later in life could be that boys are more likely to engage in rough-and-tumble play and play fights [43], and are thus desensitized to physical abuse to a higher extent than women. It is also, however, possible that measurement invariance could explain the perceived gender differences.

Our current findings suggest that, fortunately, more than half of the participants who have severe experiences of abuse and neglect in childhood seem to succeed in overcoming some of the possible consequences with regards to depression and anxiety symptoms and alcohol abuse in adulthood. While the present study did not investigate mediators of resilience, many studies have considered successful psychosocial adjustment as a mediator of psychological resilience following adverse events [44–45]. It should also be mentioned that some individuals likely have heritable factors that have been shown to protect against adverse effects of maltreatment, by means of gene–environment interaction (i.e., the concept that individuals respond differently to environmental stressors depending on their genotype) [46].

### Limitations of the research

Despite the strengths of the present study, it is also characterized by some limitations worth mentioning. First, memories are usually influenced by later experiences, and since the questionnaire was about events that happened during childhood, the obtained information might be somewhat biased. Second, we did not consider the possible overlap between experiences of maltreatment types. Because experiencing one type of abuse or form of neglect is associated with experiencing also another type of abuse or form of neglect [10, 47], it is possible that also severe forms of abuse and neglect are correlated across types or maltreatment. This could, for example, mean that several of the individuals with clinical cases of depression and anxiety or alcohol abuse, not only had experienced one form of severe abuse, but several. Should this be the case, the additive effect of multiple types of abuse could influence the results.

In the present study, it is possible that the true prevalence of anxiety, depressive symptoms or alcohol abuse has been underestimated, as we have only one cross-sectional assessment of the above mentioned indicators (i.e., some individuals may have experienced clinically significant symptoms before study participation, or may experience symptoms in the future, but did not do so at the time of assessment). A longitudinal assessment of adulthood symptoms would thus arguable have been more appropriate than a single, cross-sectional measure.

Also, some of our results and group comparisons were based on very few individuals. This might both influence the estimated prevalence of depression and anxiety or problematic alcohol use and undermines the statistical power to detect differences. Finally, we only included three known consequences of experiencing childhood maltreatment: Depression and anxiety and problematic alcohol use. It is possible that individuals showing resilience on these possible consequences of maltreatment are not resilient with respect to other negative outcomes, such as social functioning or health-risk behavior.

## Conclusions

To our knowledge, this is the first study that has looked at the effects of severe experiences of abuse in childhood on depression and anxiety symptoms and alcohol abuse in adulthood in a relatively large sample. We found that a majority of individuals with severe experiences of childhood maltreatment did not meet the criteria for clinical of levels depression and anxiety or clinical significant levels of alcohol abuse. Although this is a positive message, it is important to remember that experiences of child maltreatment increase the risk of psychosocial problems in adulthood and several of the victims of severe maltreatment included in our study may have had increased, but non-clinical significant levels of depression, anxiety, and alcohol abuse.

## Supporting information

S1 Dataset SPSS sheet containing severe experiences of childhood maltreatment and depression, anxiety and alcohol abuse data. Please refer to the SPSS variable list for variable descriptions.
(SAV)
