# Correction: Experiences of severe childhood maltreatment, depression, anxiety and alcohol abuse among adults in Finland

**DOI:** 10.1371/journal.pone.0180316

**Published:** 2017-06-22

**Authors:** The PLOS ONE Staff

The headings are incorrectly placed in Table 3. Please see the corrected Table 3 here. The publisher apologizes for the error.

**Table 3:** Differences between men and women in severe experiences of different types of maltreatment on clinical cases of depression and anxiety.

Severe Abuse		All Participants				Men				Women			
		Depression or Anxiety				Depression or Anxiety				Depression or Anxiety			
		Yes (n)	No (n)	Χ^2^	OR (95% CI)	Yes (n)	No (n)	Χ^2^	OR (95% CI)	Yes (*n*)	No (n)	Χ^2^	OR (95% CI)
CTQ Emotional Abuse	Yes (*n*) No (*n*)	19 1091	45 9825	27.154***	3.80 (2.22, 6.52)	4 439	5 3318	9.28**	6.05 (1.62, 22.60)	15 652	40 6507	21.46***	3.74 (2.06, 6.81)
CTQ Physical Abuse	Yes (*n*) No (*n*)	8 1102	18 9852	12.241***	3.97 (1.72, 9.16)	4 439	5 3318	9.28**	6.05 (1.62, 22.60)	4 663	13 6534	4.14*	3.03 (.99, 9.33)
CTQ Sexual Abuse	Yes (*n*) No (*n*)	8 1102	35 9835	3.428	2.04 (0.94, 4.41)	0 443	2 3321	0.27	n.c	8 659	33 6514	5.18*	2.40 (1.10, 5.21)
CTQ Emotional Neglect	Yes (*n*) No (*n*)	12 1098	32 9838	14.321***	3.36 (1.73, 6.54)	0 443	7 3316	0.94	n.c	12 655	25 6522	23.83***	4.78 (2.40, 9.56)
CTQ Physical Neglect	Yes (*n*) No (*n*)	4 1106	3 9867	17.051***	11.90 (2.66, 53.22)	1 442	0 3323	7.50**	n.c	3 664	3 6544	11.89***	9.86 (1.99, 48.93)

Note: Odds Ratios above 1 indicate higher prevalence of depression and anxiety in individuals experiencing severe forms or maltreatment.

*** *p* < .001

** *p* < 0.01

*p < .05, n.c. = not calculable due to at least one cell having zero observations
